# A Rare Extrahepatic Biliary Anomaly

**DOI:** 10.1155/1989/49560

**Published:** 1989

**Authors:** A. Elhamel

**Affiliations:** Alfateh University P.O Box 3704 Tripoli Libyan Arab Jamahiriya

## Abstract

In a case operated on for calculous jaundice the right and left hepatic ducts drained directly into the gallbladder and the cystic duct was the only route by which hepatic bile reached the duodenum. Exploration of the inside of the gallbladder was a crucial step for the discovery of the anomaly. Continuity of bile drainage was secured by the preservation of a portion of the gallbladder which contained hepatic and cystic ducts.


*HPB Surgery* 1989, Vol. 1, pp. 353–358
Reprints available directly from the publisher
Photocopying permitted by license only

© 1989 Harwood Academic Publishers GmbH
Printed in Great Britain

### CASE REPORT
### A RARE EXTRAHEPATIC BILIARY ANOMALY

A. ELHAMEL

*Associate Professor of Surgery, Alfate^ch University*

*(Received 16 June, 1988)*

In a case operated on for calculous jaundice the right and left hepatic ducts drained directly into the gallbladder and the cystic duct was the only route by which hepatic bile reached the duodenum. Exploration of the inside of the gallbladder was a crucial step for the discovery of the anomaly. Continuity of bile drainage was secured by the preservation of a portion of the gallbladder which contained hepatic and cystic ducts.

KEY WORDS: Bile duct anomaly, cholecystectomy.

### INTRODUCTION

The common bile duct is the least variable segment of the biliary tree and its congenital absence is the rarest recorded anomaly of the extrahepatic biliary system[1]. This anomaly was first described in 1871 by Crucknell, and a review of the literature reveals only nine documented cases reported so far. In congenital absence of the common bile duct hepatic bile enters the gallbladder via cholecystohepatic ducts and reaches the duodenum through the cystic dict. To prevent discontinuity of biliary drainage into the duodenum, therefore it is necessary to recognise this anomaly at operation and not to remove the gallbladder in its entirety. The encounter of an absent choledochus during cholecystectomy in one patient prompted this report with a brief review of the literature.

### *Case Report*

A 55 year-old woman was admitted to the Tripoli Central Hospital in February 1986 with complaints of right upper abdominal pain of 1 year duration and recent discolouration of the skin. Clinical and laboratory investigations confirmed the presence of jaundice, and an abdominal ultrasound investigation demonstrated multiple stones in the gallbladder. At operation a thick-walled gallbladder containing multiple stones was found. It was also noted that the gallbladder was located much closer to the midline than normal, with its body densely adherent to the liver hilum. Because of this peculiarity an intraoperative cholangiogram was carried out through the cystic duct. The x-ray showed a gallbladder full of stones and a long tortuous cystic duct draining into the duodenum. There was no evidence of the common bile duct. The gallbladder was opened and stones were extracted. Inspection of the inside of the gallbladder revealed the presence of two small openings located in the supero-posterior portion of the gallbladder which was

Correspondence to: A. Elhamel, P O Box 3704, Tripoli, Libya adherent to the liver hilum. A probe introduced in each opening, led into the right and left liver lobes respectively. To ensure continuity of bile drainage into the duodenum only the accessible portion of the fundus and body of the gallbladder was excised. The remaining gallbladder was repaired after the insertion of a tube. The postoperative course was uneventful. A tube cholangiogram performed on the 9th postoperative day demonstrated the anatomical arrangement observed at operation (Figure 1) the right and left hepatic ducts drained directly into the gallbladder and the cystic duct was the sole route by which bile reached the duodenum. The patient was discharged on the 10th postoperative day with normal liver function tests. She remains asymptomatic and free of jaundice 2 years after her operation.

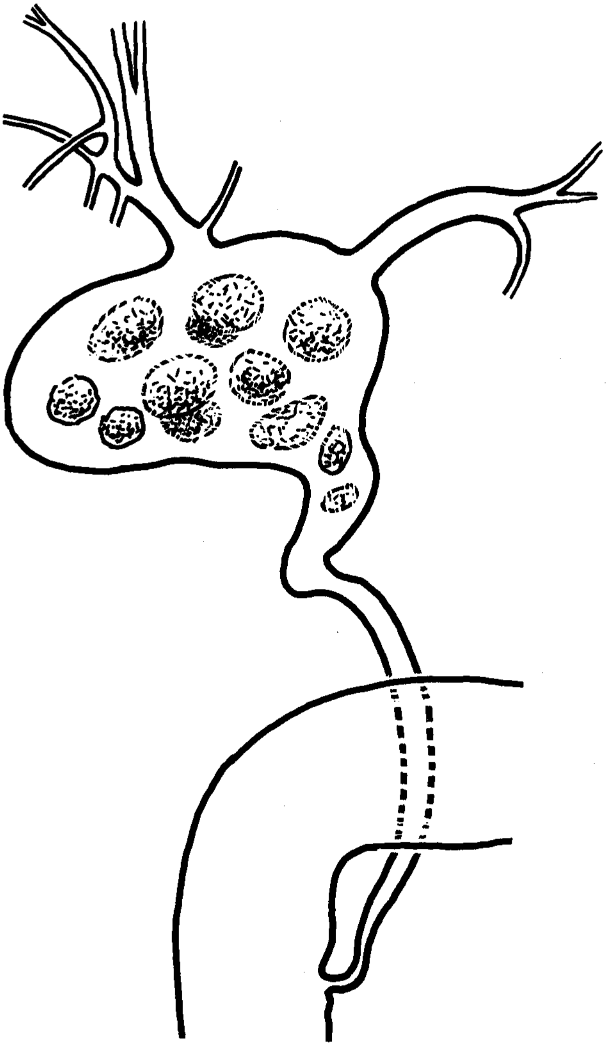

**Figure 1a** Diagrammatic representation of the anomaly as observed at operation

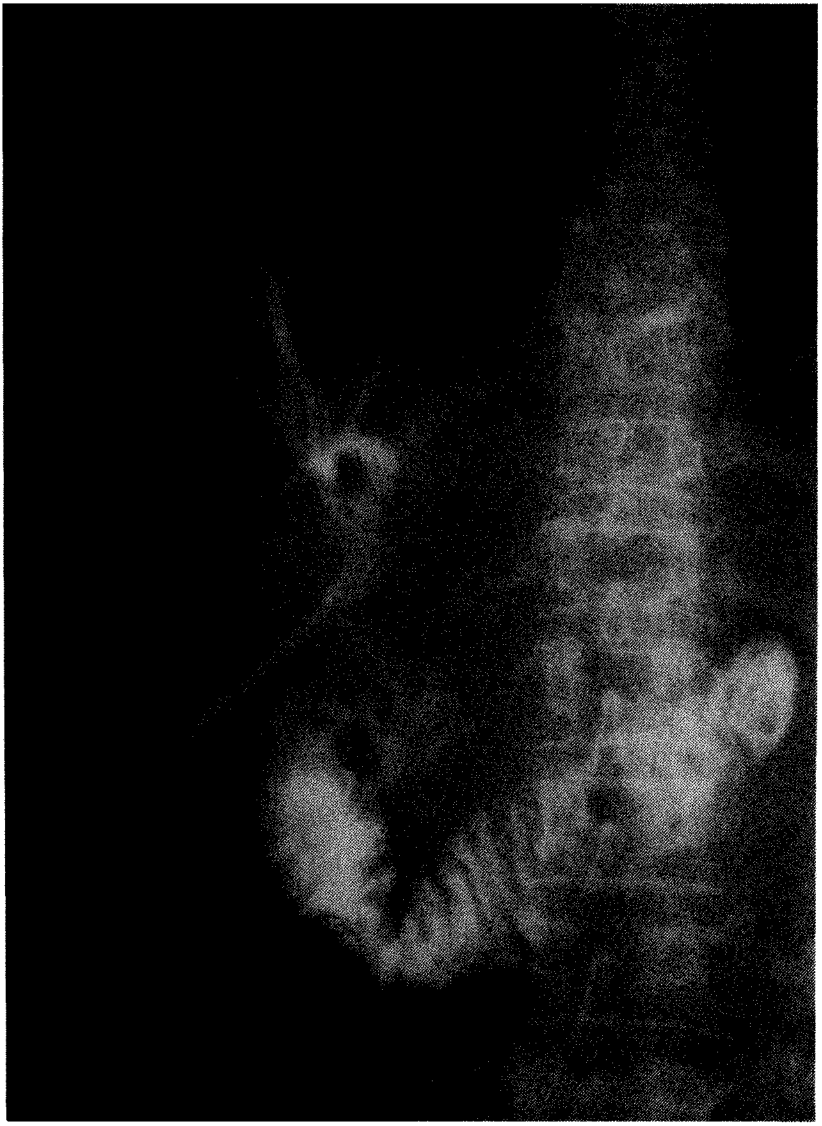

**Figure 1b** Tube cholangiogram demonstrating the abnormal anatomical arrangement of extrahepatic biliary tract.

*Comment*

Congenital absense of the common bile duct has been described in nine instances in the world surgical literature (Figure 2). In seven cases the anomaly was observed in patients undergoing cholecystectomy for cholelithiasis or cholecystitis, and in two it was reported in autopsy specimens[2,3]. The first authentic case recorded was that

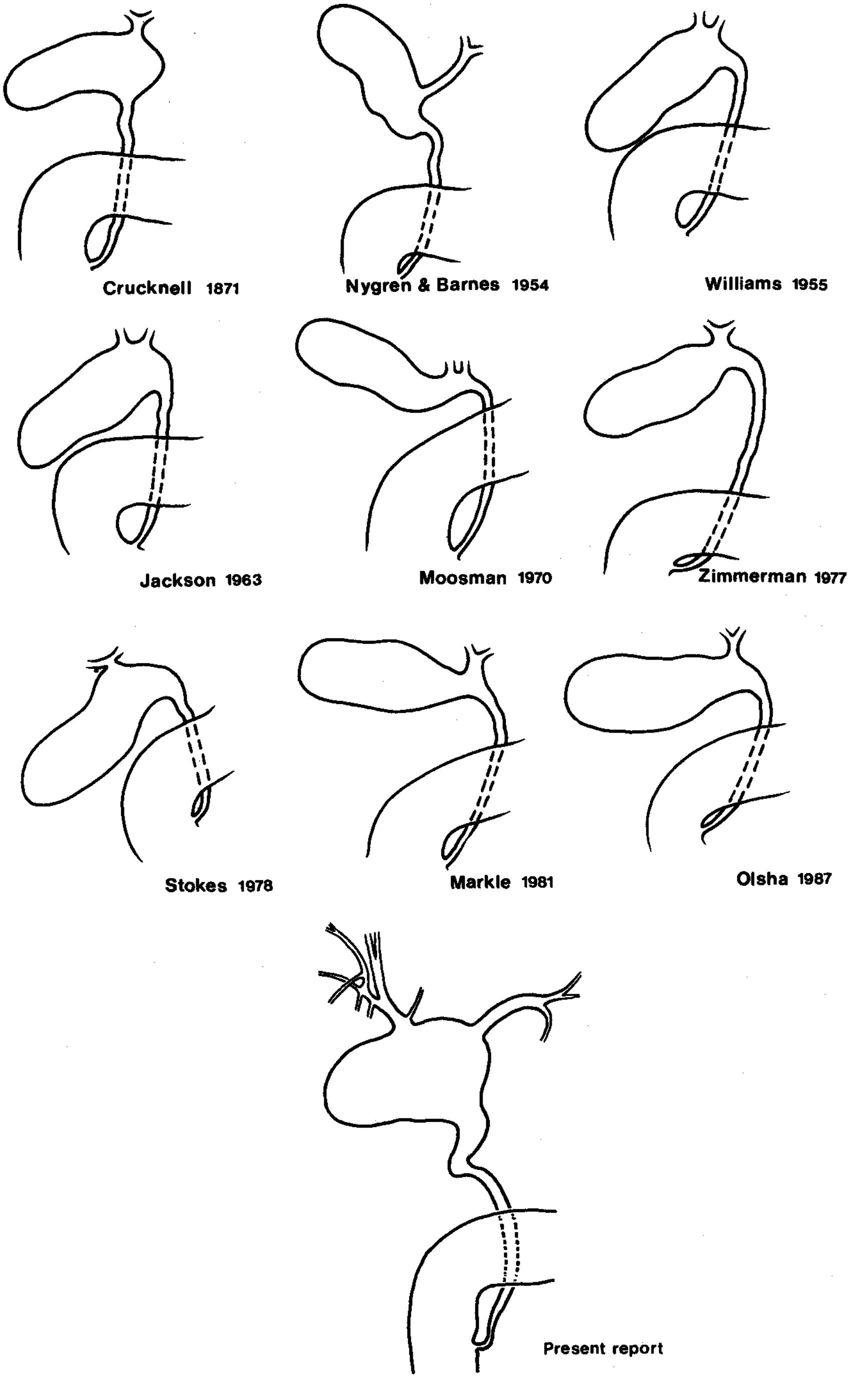

**Figure 2** Schematic representation of the anomaly in the reported cases.

of Crucknell (1871) who stated that in a specimen taken from the body of a man the cystic duct was the sole channel of communication between the liver and the duodenum[2] In 1954 Nygren and Barnes[4] described the first surgical case of absence of the common bile duct encountered during cholecystectomy. Since then six other instances have appeared in isolated published reports [5–10]. The present report brings the total number of cases to ten.

Diagnosis of the anomaly was made on operative and radiological findings, histologic examination being referred to only in one of the reported surgical cases[8]. It may be argued that in the absence of histological confirmation the diagnosis may be that of an absent gallbladder with circumscribed cystic dilatation of the proximal bile duct. Co-existence of these two rare anomalies, to my knowledge has not been reported in the available literature. Moreover, absence of the gallbladder, as commented on by many authors, is not usually accompanied by any dilatation of bile ducts and is observed in about one-sixth of atresia of the extrahepatic bile passages[11]. The duct which emerged from the gallbladder is generally considered to be the anatomical cystic duct rather than a common bile duct with interposition of the gallbladder between it and the hepatic duct or ducts[9]. This unique arrangement of bile drainage is normal in certain fishes and some other lower animals[12]. The embryological explanation of this anomaly is attributed to failure of recanalization of hepatic ducts and persistence of fetal connections as cholecysto-hepatic ducts[13].

Hepatic bile entered the gallbladder via a common hepatic duct in five of the reported cases and through the right and left hepatic ducts in three cases, including our own. Failure of initial recognition of the anomaly occured in five patients. Removal of the gallbladder inevitably resulted in discontinuity of bile drainage which was re-established at the time of operation by hepatico-duodenostomy in two patients[8,10] and by end-to-end anastomosis of the hepatic and the cystic duct or by hepatico-jejunostomy and a long trans-hepatic tube in one case each[7,9].

An unsuccessful outcome followed failure to recognise the anomaly in the fifth patient[5]. In the other two operative reports[4,6] the anomaly was recognised and a portion of gallbladder was preserved to permit closure over a cholecystostomy tube. In one of these[6], as in the present case, hepatic ducts were identified only by exploration inside the gallbladder. Co-existent inflammation and pathological changes may obscure the presence of the anomaly on initial evaluation and can make its assessment difficult. Awareness of the possibility of this anomly, facilitated by intraoperative cholangiography and/or echography should prompt careful operative exposure and avoid a disastrous surgical error.

### *References*

1. Braasch, J.W. (1958) Congenital anomalies of the gallbladder and bile ducts. *Chir J North Am.*, **38**, 627–630
2. Crucknell, H.H. (1871) Malformation of the gallbladder and hepatic ducts. *Tran Path soc Lond.*, **22**, 163–164
3. Moosman, D.A. (1970) The surgical significance of six anomalies of the biliary duct system. *Surg gynecol Obstet.*, **131**, 655–660
4. Nygren, E.J. and Barnes, W.A. (1954) Atresia of the common hepatic duct with shunt via an Accessory duct. *Arch Surg.*, **68**, 337–343
5. Williams, C. and Williams, A.M. (1955) Abnormalities of the bile ducts. *Ann Surg.*, **144**, 598–605

6. Jackson, J.B. and Kelly, T.R. (1964) Cholecysto-hepatic ducts. *Ann Surg,* **159**, 581–584
7. Zimmerman, H.G. (1977) Zwischenschaltung der Gallenblase in der ductus hepato-choledochus, eine Seltene Anomalie der Gallenwege. *Chirurg,* **48**, 73–76
8. Stockes, T.L. and Old, L. (1978) Cholecysto-hepatic duct. *Am J Surg,* **135**, 703–705
9. Markle, G.B. (1981) Agenesis of the common bile duct *Arch Surg,* **116**, 350–352
10. Olsha, O., Steiner, A., Rivkin, L.A. and Shienfeld, A. (1987) Congenital absence of the anatomical bile duct. *Act chir Scand,* **153**, 387–390
11. R.E. Grosse (1936) Congenital anomalities of the gallbladder. *Arch Surg* **32**, 131–162
12. Mentzer, S.H. (1929) Anomalous bile ducts in man. *JAMA,* **93**, 1273–1277
13. Hayes, M.A., Goldenberg, I.S. and Bishop, C.C. (1958) The developmental basis for bile duct anomalies. *Surg Gynecol obstet,* **107**, 447–456

*(Accepted by S. Bengmark on 30 November 1988)*

### INVITED COMMENTARY

This interesting paper documents the occurrence of a rare anomaly seen in one patient and successfully treated by the author. The question whether the anomaly could have been a choledochal cyst in a patient in whom a gallbladder was either absent or not found is addressed by the author. In my view this alternative explanation cannot be discounted without histology of the gallbladder wall proving that it is not a choledochal cyst. If one takes the stance that this could be a choledochal cyst then the described anomolous anatomical situation would be correct as would the finding of right and left ducts draining into the cystic structure. The "cystic duct" draining below would become the common bile duct draining below a cholecochal cyst. In addition to histology of the removed wall, an ERCP may help to confirm that the suggested diagnosis is correct.

Despite the question raised about a variant of a choledochal cyst this does not distract from an excellent short presentation documenting the tenth case reported in the literature of possible congenital absence of the common bile duct.

Professor John Terblanche
Department of Surgery
University of Cape Town
Cape Town, South Africa

